# The changing climate and human vulnerability in north-central Namibia

**DOI:** 10.4102/jamba.v8i2.200

**Published:** 2016-02-02

**Authors:** Margaret N. Angula, Maria B. Kaundjua

**Affiliations:** 1 Department of Geography, History and Environmental Studies, University of Namibia, Namibia; 2 Department of Sociology, University of Namibia, Namibia

## Abstract

North-central Namibia is more vulnerable to effects of climate change and variability. Combined effects of environmental degradation, social vulnerability to poverty and a changing climate will compromise subsistence farming in north-central Namibia (NCN). This will make subsistence and small-scale farmers in the region more vulnerable to projected changes in the climate system. Thus, the aim of this article was to examine factors contributing to subsistence farmers’ vulnerability to impacts of climate change. The article further discusses different aspects of human vulnerability and existing adaptation strategies in response to impacts of climate related disasters experienced over the past three to four decades in NCN. Qualitative and quantitative research approaches and methodology were employed to obtain information from subsistence farmers in north-central Namibia. The sociodemographic characteristics of Ohangwena, Oshana and Omusati Region reveals high levels of unemployment, high adult and elderly population and high dependency on agricultural livelihood system. These indicators help understand levels of household vulnerability. The study concludes that households interviewed revealed low levels of adaptive capacity due to exposure to climate risks and combined effects of social, political and cultural factors. This article provided an understanding that is required to inform the adaptation pathways relevant for NCN.

## Introduction

The Intergovernmental Panel on Climate Change’s (IPCC) fifth assessment report projects that global mean temperatures will continue to rise over the 21st century if greenhouse gas emissions continue unmitigated (IPCC 2014). Thus, global temperatures averaged over the period 2081–2100 are projected to likely exceed 1.5 C. This is expected to have a range of impacts on food security, human health and human security in sub-Saharan Africa. It is also widely acknowledged that the impacts are quite variable, with the poor nations bearing the most effects of climate change (IPCC 2014; Rurinda *et al*. 2014). As such, north-central Namibia (NCN) is more vulnerable to effects of climate change and variability. This is so because 57% of its rural population rely heavily on subsistence agriculture for their livelihood (Namibia Statistic Agency [NSA] 2011:8). A combined effect of environmental degradation, social vulnerability to poverty and a changing climate will compromise subsistence farming in NCN. This will make subsistence and small-scale farmers in the region more vulnerable to projected changes in the climate system. Thus, the aim of this article is to examine factors contributing to subsistence farmers’ vulnerability to impacts of climate change and related risks. The article further discusses different aspects of human vulnerability and existing coping strategies in response to observed impacts of climate related disasters experienced over the past three to four decades in NCN.

### Literature review

#### Conceptualising vulnerability

The definitions and assessments of climate change vulnerability are often applied inconsistently (Shah *et al*. 2013). There seems to be slight variations in the understanding of climate change vulnerability amongst social sciences and natural sciences scientists. The fourth IPCC assessment provides a useful typology suggesting that vulnerability may be a function of adaptive capacity, sensitivity and exposure (Schneider *et al*. 2007). Vulnerability is defined in the fifth IPCC assessment as the pronspensity or predisposition to be adversely affected. This definition also encompasses a variety of concepts including sensitivity or susceptibility to harm and lack of capacity to cope and adapt. In a process of understanding vulnerability various authors have contributed significantly to the conceptualisation of climate change vulnerability also highlighting the social and livelihood vulnerability aspects. Contextualising vulnerability in the climate change discourse, Kelly and Adger (2000) and O’Brien *et al*. (2007) clarified this concept by differentiating between ‘end-point’ and ‘starting-point’ features of climate change vulnerability. The IPCC’s fifth assessment referred to these concepts as ‘outcome vulnerability’ and ‘contextual vulnerability’; respectively (IPCC 2014). Whilst the outcome vulnerability (end-point) refers to consequences of analysis beginning with projections of future emission trends, the current study build on the concepts of contextual vulnerability (starting-point) which refers to present inability to cope with external pressures or changes such as changing climate conditions. Contextual vulnerability is a characteristic of social and ecological systems generated by multiple factors and processes (IPCC 2014; O’Brien *et al*. 2007).

#### Climate change vulnerability in Namibia

The climate in the study area is semi-arid and characterised by highly variable climatic conditions and seasonal rainfall falling mostly from November to April (Mendelsohn, El Obeid & Roberts 2000). Consequently, reoccurring droughts, heavy rainfall events, episodes of higher temperature and unpredictable and variable rainfall have been experienced in the past 30 to 40 years in Namibia (Kaundjua, Angula & Angombe 2012; Newsham & Thomas 2011; Republic of Namibia 2011). The latest projections for Namibia are based on the second national communication in terms of the United Nations Framework Convention on Climate Change (UNFCCC) report (Republic of Namibia 2011). Accordingly, Namibia is projected to become hotter throughout the year with a predicted increase in temperature between 1 °C and 3.5 °C. The northern and central regions of Namibia have observed changes highly variable and spatial heterogeneity in rainfall patterns. More variable pattern of rainfall is predicted for Namibia therefore climate change will cause increased aridity due to the combined effect of variable rainfall and increased evaporation (30%) by 2020. As a result, rainy seasons are expected to be shorter and rainfall is likely to increase over much of Namibia. The projections suggest that key sectors that support Namibia’s economy and food security are most vulnerable (Dirkx *et al*. 2008). It is generally believed that the changing climate will have an adverse effect on the agricultural yield directly through changes in temperature and precipitation, and indirectly through changes in soil quality, pests, and diseases. Flash floods are further predicted to impact overall sanitation and human health conditions (Kaundjua *et al*. 2012).

Several studies revealed that climate change vulnerability increases existing vulnerability of rural livelihoods and reduces household adaptive capacity (Heltberg, Siegel & Jogernsen 2009; Kaundjua *et al*. 2012; Shah *et al*. 2013). This is due to their differential levels of exposure to climate risks as well as their limited adaptive capacity. The combination of climate related impacts and nonclimatic drivers also increase contextual vulnerability of rural subsistence farmers in Namibia as in the rest of Africa. Cutter *et al*. (2000) cited in Shah *et al*. (2013:2) argues that social vulnerability is partly a product of these factors. Therefore livelihood and social vulnerability to climate change can be best understood as an outcome of climatic and nonclimatic drivers that influences human and natural systems. The vulnerability of climate change in Namibia is differentiated spatially (between regions) and socially (amongst social groups e.g. gender, class, marginal groups and ethnicity). Angula and Menjono (2014) and Kuvare, Maharero and Kamupingene (2008) reported that the social network and support system in NCN is declining.

There is limited literature on vulnerability indicators and assessment in Namibia. What is known are the factors and determinants of vulnerability in Namibia. The Republic of Namibia (2011) identified biophysical determinants of vulnerability for the agricultural sector as rangeland and grass availability for livestock rearing, water availability and demand, disease and pests impact on crop and livestock health. Thuiller *et al*. (2006) and Dirkx *et al*. (2008) project significant changes in the vegetation structures. Reductions in vegetation cover and Net Primary Productivity (NPP) have negative implications for grazing. Furthermore, increased temperature effects on conception in cattle have impact on livestock breeds that are not adapted to higher temperatures (Republic of Namibia 2011). Zeidler *et al*. (2010), Angula (2010) and Kuvare *et al*. (2008) stated that subsistence farmers have noted impacts of observed climate changes on the quality of soil and productivity of agricultural arable lands in NCN. Primary impacts of observed climate changes on land productivity had led to a decline in crop yields. The reduction in crop production in the NCN is projected to decline by 50% (Republic of Namibia 2011:76). The secondary impacts of reduced crop yield will result in decreased household food security, increased poverty and increased rural – urban migration for subsistence farming communities in NCN.

## Research methodology

This study draws on both qualitative and quantitative approaches to identify existing vulnerabilities of subsistence farmers in NCN to the impacts of climate change. Furthermore the approaches aimed to determine existing adaptation strategies applied in farmers in response to climate related disasters. This study used the 2008–2012 floods and droughts experienced over the three decades to understand vulnerability of subsistence farmers. The research approaches were guided by the climate change vulnerability and capacity assessment (CVCA) framework and socio vulnerability assessment approach.

**FIGURE 1:**
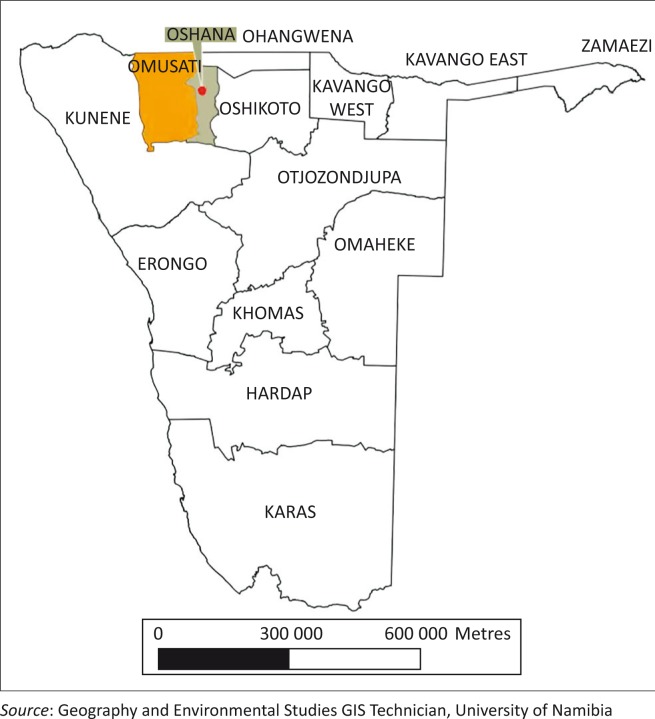
A map illustrating 14 administrative regions of Namibia.

The fieldwork was carried out amongst selected communities in three of the NCN regions, namely Ohangwena, Oshana and Omusati. These regions are located in the extreme northern parts of Namibia bordering Angola (Figure 1). The NCN regions host the Owambo ethnic groups which comprise the majority (40%) of the Namibian population (Namibia Statistics Agency 2011:27). Ohangwena and Oshana regions are the most densely populated regions in the country, with 22.9 and 20.4 people per square kilometres, respectively (Namibia Statistics Agency 2011:32). The subsistence farmers in NCN rely on rain-fed crop and livestock production for their livelihood.

The fieldwork was carried out in a sequence of two phases. Phase 1 focussed on the collection of qualitative data using focus group discussions and in-depth interviews. The first phase (April 2011) concentrated mainly on the communities from selected informal settlements in the outskirts of Oshakati town whose locations were submerged by floodwater and were evacuated as well as rural villages in Oshana and Ohangwena who were affected by floods during 2011 but were not evacuated. For the qualitative approach, Focus Group Discussions (FDG) and key-informant interviews were conducted amongst communities mentioned above, in order to get an in-depth understanding of social vulnerability dynamics. The selection to these communities was based on the fact that it is important to identify differentials in the perceptions of climate change impacts, vulnerability, adaptation and coping strategies amongst various social groups.

Quantitative data were collected during phase 2 (June/July 2012) using household survey questionnaires administered to household heads in selected villages in Ohangwena, Oshana and Omusati region. A household head was defined as a person of either sex who was looked upon by other members of the households as their leader or main decision-maker during fieldwork (Namibia Statistic Agency 2011). The questions in the household survey questionnaire were mainly close-ended with a few open-ended questions focussing on the identification of household livelihood, the occurrence and intensity of natural disasters, as well as the disaster impact on their livelihood. In addition, the household survey questionnaire included questions on the impact of the climate related natural disasters on population displacement and movements and impacts of the climate related natural disasters on human health (Table 1).

**TABLE 1:** Number of household survey questionnaires completed, focus group discussions held and key-informant interviews conducted during phases 1 and 2.

Region	Household survey questionnaires structured interviews	Focus group discussions	Key-informant interviews
Ohangwena	100	5	4
Oshana	101	5	4
Omusati	100	5	5
**Total**	**301**	**15**	**13**

Hence, Focus Group Discussions, key-informant interviews and household survey questionnaires were conducted with men and women from the following villages:

- Ohangwena region – Oimbandalunga and Onghala
- Oshana region – Oshaandja and Oniimwandi
- Omusati region – Ombandjele, Oshondo, Omuyala.

Whereas quantitative data were analysed using SPSS, qualitative analyses focussed on mapping out emerging issues from FGDs and in-depth interviews. The analyses were complemented by findings from national and research reports. Results from both approaches were interpreted together in order to complement each other and also to cancel out the weaknesses of each research approach (quantitative and qualitative).

## Findings

### Sociodemographic characteristics

The findings from this study are consistent with other studies revealing that the majority of households in rural NCN are either female headed or female de facto headed households. Approximately 79% of the households head respondents from Ohangwena and Oshana regions were females, whereas 60% of the respondents in Omusati region were males. The majority of the respondents from Ohangwena and Oshana were either aged between 40 or 59 years old (43%) or older than 60 years (39%). Omusati region followed the same pattern with 48% and 27% for adults and elderlies respectively. Only 16% of respondents obtained tertiary education; the rest of the respondents have either achieved secondary education (21%) or primary education (54%).

As discussed earlier the study area is mainly occupied by subsistence farmers. It is thus not surprising that only 13% of the respondents were formally employed. The households were also asked to indicate their monthly income from various sources. In the majority of the households, heads (80%) earned less than R1000.00. Consequently, the main sources of livelihood are livestock and crop farming (Table 2).

**TABLE 2:** Main source of livelihood.

Source of livelihood	Number of responses	%
Crop production	174	57.8
Livestock farming	15	4.9
Informal business	11	3.7
Employment (salary)	39	12.9
Remittances	5	1.6
Pensions or social Grants	52	17.2
No response	5	1.6
**Total**	**301**	**100**

Respondents were further asked to indicate the second and third most important source of livelihood. Livestock farming (37%) was mentioned as the second most important source of livelihood followed by pension or social grants (15%). Land tenure in the rural NCN is communal land. Under this land tenure, communities share natural resources, rangeland and water resources for both household and livestock consumption. However, each household is allocated an individual plot for crop cultivation under the right of lease hold system.

With regard to health, the health system and service delivery in the rural NCN are mainly dominated by the public services sector which is based on the primary health care approach (Namibia Demographic and Health Survey 2008). Malaria, HIV and/or AIDS, tuberculosis and malnutrition are the main health problems causing ill-health and deaths in NCN (Namibia Demographic and Health Survey 2008). This may have implications for the subsistence agriculture sector in NCN because substantial amount of agricultural labour are lost.

### Impacts of climate variability and change on livelihood system

The findings of this study are consistent with Angula (2010) in that subsistence farmers’ (from NCN) definition and perception of climate change (as a concept) does not differentiate long-term climate change, climate variability and short-term seasonal climate variability. However, participants were assessed on their knowledge of climate change and whether they distinguish natural climatic variability from UNFCCC climate change. The perception and views of local people on climate change are closely linked to observed changes over the past 2 to 3 decades. During FDG and key informants in-depth interviews, subsistence farmers were asked to recall changes in the temperature and rainfall. Respondents were further asked to describe the primary and secondary impacts of these changes on the main sources of livelihoods (livestock and crop farming). Table 3 (based on findings from FGDs and key-informant interviews) presents observed changes, primary impacts and secondary impacts of climate change on subsistence farming.

**TABLE 3:** Observed changes and impacts on subsistence agriculture.

Observed changes	Primary impacts†	Secondary impacts‡
Late arrival, early withdrawal of rainfall	Reduced agricultural crop yield	Men migrate in search for better grazing opportunities or employment opportunities
Irregular dry spells coinciding with critical growing stages of crops and vegetation	Reduced grazing area and overgrazing	Increased poverty
Days are getting hotter	Reduced number of livestock	Hunger and famine (nutrition deficiency and malnutrition)
Changes in wind intensity and direction	Reduction in land productivity	Poor boreholes or wells recharge
Increased rainfall and flooding (2008–2011)	Declining quality of soils for sorghum and *mahangu* farming	Destruction of properties
Increased drought incidences	Loss of land productivity and soil degradation	Restriction of access to and disruption of services
-	Lack of construction materials Increased water shortages for livestock consumption	Disruption of family structure (evacuation camps)
-	Pests outbreak destroying crops	Threat to physical health (waterborne and vector diseases)
-	Disease and parasites affecting livestock	-

† Primary impacts in this article refers to direct impacts on the natural and livelihood systems due to climate variability and change;

‡ Secondary impacts in this article refer to indirect impacts resulting from primary impacts due to climate variability and change.

**FIGURE 2:**
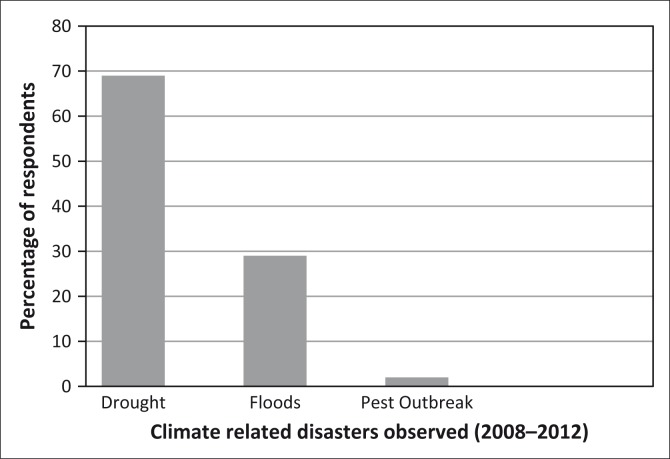
Climate related disasters observed in North-Central Namibia (2008–2012).

During the household survey respondents were asked to recall experienced climate related disasters of floods and droughts during 2008–2012 (Figure 2). These disasters are associated with impacts listed in Table 3. These disasters also increase livelihood and household vulnerability of subsistence farmers in NCN.

The results of the study based on the perceptions of respondents, show that they viewed flood intensity to be severe compared to drought (Table 4). The community from Ohangwena and Oshana regions indicated that the severity of drought were average (33%) compared to Omusati region that reported that droughts are not at all intense (100%).

**TABLE 4:** Intensity of drought and floods experienced during the period between 2008 and 2012.

Climate risk	Regions	Severe (%)	Mild (%)	Not at all (%)
Drought	Ohangwena and Oshana regions	3	33	64
	Omusati region	0	0	100
Floods	Ohangwena and Oshana regions	98	2	0
	Omusati region	89	11	0

As discussed in the literature review and consistent with qualitative research findings, 48% of the respondents stated that destruction of properties and disruption of services, threat to physical health (40%), increased poverty (10%) and disrupted family structures (2%) are amongst secondary impacts experienced due to the changing climate.

**FIGURE 3:**
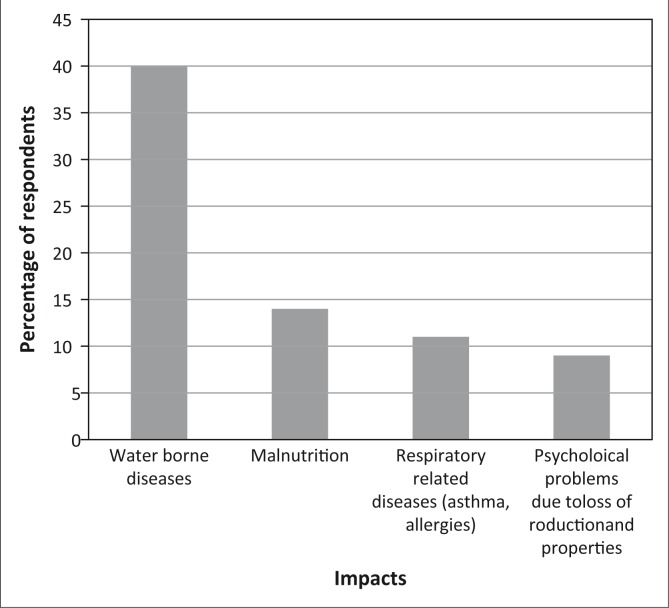
Impacts of flooding on human health.

The study also assessed migration and health impacts of climate change in the study area. In Ohangwena and Oshana regions respondents (87%) moved or migrated due to flooding between 2008 and 2012. Due to the ongoing rural-migration trends in the study area, climate related disasters add to the existing stressors causing emigration. Correspondingly, 47% of the respondents moved due to temporary emergency evacuation followed by employment and educational purposes (35%). Only 18% of the household members moved due to seasonal grazing. The respondents from Omusati region perceive that impacts of flooding had health related effects on people (Figure 3).

### Human vulnerability amongst subsistence farmers

Previous studies revealed that crop and livestock farming followed by social grants, employment and remittances make up the livelihood system in NCN. Similarly, Table 2 (previous subsections) illustrates that crop production is the main livelihood strategies in all three regions. Social grants and employment followed and livestock production are ranked as the fourth most important livelihood strategies. Men and women participants in FDGs identified households that have no access or limited access to social grants and cash income to be more vulnerable because of the higher dependency on climate-fed crop and livestock subsistence farming. Furthermore, Angula and Menjono (2014) argued that social vulnerability is differentiated by gender, age and social status. In Ohangwena region, it emerged from in-depth interviews that community members are worried about their livelihood as they felt it is badly affected by frequent floods and drought experienced in the past five years. Subsistence farmers expressed their fears (during FGDs) that climate change may add additional burden to the government and non-governmental organisations (NGOs) because of additional budget required to increase drought relief. FGDs participants felt that developmental projects will get delayed as finances are diverted into disaster risk response and renovation of infrastructure efforts. Informal business was mentioned as one of the livelihood strategies that complement farming. FGDs participants from Oshana region reported that in event of flooding, business is disrupted affecting cash income for most households. This cash income is mainly used to pay education and health related costs, improve agricultural input and pay for household and livestock water consumption bill. Finally, the 2011 flood left roads and infrastructure in Oshana region destroyed thereby limiting agricultural extension officers in providing services to subsistence farmers in Ohangwena, Oshana, and Omusati regions.

### Existing adaptation and coping strategies employed by subsistence farmers in north-central Namibia

In response to impacts of floods and droughts experienced in NCN, subsistence farmers interviewed recounted that at household level they engage in short-term adaptation strategies in response to impacts of changing climate and with the aim to reduce contextual vulnerability. Table 5 lists adaptation options employed by subsistence farmers from Ohangwena, Oshana and Omusati Regions.

**TABLE 5:** Existing adaptation options in North-Central Namibia.

Climate exposure	Adaptation option
Increased rainfall (flooding)	Male migrate to areas not affected by floods (livestock)
	Move into evacuation camps (severe floods)
	Dig trenches in and around crop fields to reduce inundation
	Supplement agricultural based livelihood with cash income or remittances or pension
	No long-term adaptation options identified in the study
	No rainwater harvest was mentioned during the study
Reduced rainfall and extreme temperatures (drought)	Reduce impact on livestock using destocking method
	Sell livestock and purchase food to enhance food security
	Use crop variety that is resistant to drought (okashana varieties no. 1 and 2)
	Migrate to remote rangeland areas for better grazing
	Government disaster response – drought relief
	Supplement agricultural based livelihood cash income or remittances or pension
	No long-term adaptation options identified in the study

Considering that floods and drought both reduce agricultural outputs, it is not surprising that farmers adopt similar adaptation strategies as illustrated in Table 5.

In summary, subsistence farmers from the study area perceive that rainfall variability affect crop production the most. On the other hand, livestock production is perceived to be mostly affected by drought and high temperatures. These perceptions are in agreement with the vulnerability and assessment study findings as reported in the second national communication to UNFCCC (Republic of Namibia 2011). Farmers opined that existing adaptation options are insufficient to cope with more frequent episodes of drought and floods. This study reveals that there are no local climate change adaptation institutions in villages that participated in the study. The on-desk document review revealed that households are encouraged to diversify livelihoods beyond agricultural strategies. However, this study found that households from study areas are not yet diversifying their livelihood. Subsistence farmers in NCN did not report to have the capacity to manage risks by planning and investing in the future. These efforts are hindered by an Early Warning System that does not provide long-term climate projections beyond seasonal forecasting.

## Discussion

The assessment of impacts of changing climate on existing livelihood and findings from related studies and assessments (Angula & Menjono 2014; Dirkx *et al*. 2008; Kaundjua *et al*. 2012; Kuvare *et al*. 2008; Newsham & Thomas 2011; Zeidler *et al*. 2010) demonstrates that the changing climate is already affecting the subsistence farming livelihood system and human well-being in NCN. Future projections of climate change under business as usual indicate an increased in climate related events that are already experienced. This article, by focussing on drought and floods events experienced during 2008–2012, provides an insight and understanding of the level of intensity of impacts at household level as well as implications for the contextual vulnerability. Heltberg *et al*. (2009) found that risks associated with climate change could increase vulnerability unless adaptation is stepped up due to uncertainty of socioeconomic implications of climate change. Therefore unless community-based adaptation strategies are developed to enhance level of managing risks and future planning, households in NCN are not able to cope and adapt to projected impacts of climate change in rural Namibia.

This article presented factors and determinants of contextual vulnerability at household level in NCN. These factors and determinants intersect with underlying causes of vulnerability such as policy related factors. Insufficient data and general understanding of poverty, sustainable development and climate change linkages at regional and local levels hinders participation and implementation of climate change adaptation strategies that are already piloted in selected villages of NCN. Shah *et al*. (2013) suggested that understanding household vulnerability is essential for management panning in areas with limited resources and access to reliable data. This article determines that the following factors are amongst the causes of underlying vulnerability to climate change in NCN:

- over dependent on climate-fed livelihood system
- poor early warning systems for rainfall and temperature forecasts, limited access to reliable information and eroded agro-ecological indigenous knowledge
- low cash income to strengthen failing farming systems
- poor land tenure system without collateral options
- poor rangeland management characterised by uncontrolled livestock carrying capacity
- existing levels of poverty
- weakening social networks and capital
- low capacity to diversify livelihood beyond agricultural farming systems.

## Conclusion and recommendations

The sociodemographic characteristics of Ohangwena, Oshana and Omusati Region reveals high levels of unemployment, high adult and elderly population and high dependency on agricultural livelihood system. These indicators help understand levels of household vulnerability. The levels of household vulnerabilities are not homogenous and are characterised by differential state of wealth and resource endowment, land productivity, access to agricultural inputs as well as access to early warning information systems. The effects of 2008, 2009 and 2011 floods illustrated that the subsistence farming system even in communities where agro-ecological knowledge is strong, remains vulnerable to extreme climate related events (Amadhila *et al*. 2013; Newsham & Thomas 2011). When such events strike, the findings of this study are consistent with Kuvare *et al*. (2008), Newsham and Thomas (2011), and Angula (2010) who reported that farmers resort to a number of short-term adaptation strategies (Table 5). The study concludes that households interviewed revealed low levels of adaptive capacity due to exposure to climate risks and combined effects of social, political and cultural factors.

This article recommends that future research apply a combined CVCA and livelihood vulnerability indices to contextualise vulnerability assessments at local and household levels. Further research is required to develop scenarios based on past historical climatic variability and future projections of climate change in assessing community-based climate change adaptation at household and individual levels. Finally, the article recommends that adaptation pathways (Republic of Namibia 2011; Rurinda *et al*. 2014) that are relevant for NCN should include or address the following:

- identification of both short-term, medium-term and long-term strategies for the community-based adaptation framework in NCN
- scaling-up of piloted adaptation options for the crop and livestock farming system in NCN
- employ a participatory approach to community-based adaptation planning
- include the following options suggested by subsistence farmers in NCN:
  ▪ livestock that are adaptable to harsh climatic conditions
  ▪ agricultural extension support to provide fertilisers and other required agricultural inputs
  ▪ move from disaster risk response towards disaster risk preparedness in addressing effects of flood impacts.

This article reiterates Angula (2010) that more gender disaggregated data and research are required to assess communities’ existing adaptive capacity in dealing with long-term impacts of climate change.
